# Hints and Improvements in the Practice of Medicine and Surgery

**Published:** 1799-10

**Authors:** 


### HINTS AND IMPROVEMENTS IN THE PRACTICE OF MEDICINE AND SURGERY.

### *To the Editors of the Medical and Physical Journal.*

GENTLEMEN,

WITH the view of rendering as public as possible the easy means I have discovered of speedily subduing a very common, and, if treated in the common mode, a very dangerous disease, I avail myself of the extensive circulation of the "*Medical and Physical Journal*," and inclose the copy of a letter which I have this day written to His Royal Highness the Commander in Chief, on the "Prevention and Cure of Dysentery." The utility of the treatment I recommend is demonstrated by successful experience in a multitude of cases. It is consequently independent of any theory. It was, however, deduced from one simple as itself, and which, in its turn, it serves to support.

At some future period, when professional avocations are fewer and less pressing, I intend to submit to the public a more particular account both of my theory and practice, accompanied with a schedule, on a perfectly new plan, of the cases I have treated.

Wishing success to your valuable Journal,

H. M. S. *Atlas*, in TORBAY, 10*th of Aug.* 1799.

I am, Gentlemen,

Your most obedient, humble servant,

D. WHYTE, M. D.

### *Copy of a Letter to His Royal Highness the* DUKE *of* YORK.

10*th Aug.* 1799.

*May it please your Royal Highness*,

AS in the intended expedition much sickness must from fatigue and exposure necessarily ensue—and as, in the present season of the year, it is probable dysentery will be one of the most prevailing complaints, I conceive it a duty which I owe my country, to communicate to you a very simple method by which that otherwise dreadful disease may be, in a few hours, completely removed.

On

On the inſtant of attack let the belly of the patient be inveſted with five, ten, fifteen, or, if fewer will not ſuffice, twenty folds of a flannel bandage, whoſe breadth is from ſix to ten or twelve inches, or more—let the patient, moreover, be inveſted with a flannel ſhirt, or waiſtcoat with ſleeves, and immediately put to bed.—If neither flannel ſhirt or waiſtcoat can be procured, the patient may turn into bed well buttoned up in a regimental jacket. If convenient, he will alſo do well to dilute with warm gruel, while perſpiration, both general and topical, is further promoted by a covering of two, three, or four blankets, and by the excluſion of cold air, particularly partial currents.

If the purging and tormina ſtill continue, or if the patient has head-ach, or any other ſymptom of general fever, no time muſt be loſt in recurring to the lancet—and we muſt not be deterred by the low ſtate of the pulſe.—It is the removal of pain and purging that is required, and from ſucceſsful experience in ſome hundred caſes, I ſay confidently, that by ſuch means we may always ſucceed. I have frequently taken from forty to fifty and ſixty ounces of blood in a couple of hours, and in ſo doing, ſaved many valuable lives.

In moſt caſes, however, the diſeaſe will yield to flannel rollers—and it will not even be always neceſſary to put the patient to bed.

In this diſeaſe all kinds of medicine do miſchief. Wines and ſpirits are particularly injurious.

To prevent relapſes, as well as firſt attacks, expoſure to cold or moiſture, or even to agreeably-cooling currents of air, is to be carefully avoided, eſpecially when the body is warm and relaxed, as during ſleep, or after fatigue.—In ſuch circumſtances, anointing the body with oil, and wearing warm cloathing, particularly a flannel ſhirt, will be found uſeful.—I make a point of anointing all my patients on the removal of the rollers.

Among ſoldiers and ſailors ſcurvy is the moſt common pre-diſpoſing cauſe of this diſeaſe.

Scurvy is the product of nitrous or ſeptous acid gas, of which foul air or azote is the principal conſtituent, and where people are crouded together is always more or leſs preſent. Although too frequently overlooked, I have found it as common in jails and camps, as on ſhip-board during long voyages. Of the cauſe I ſay nothing.—It is too well known to require comment.

Happy ſhall I be if the above rules ſhould be acted upon, and prove the means means, as I am confident they will, of preventing the fatal consequences of one of the most dreadful maladies to which human nature is liable.

Trusting that the importance of the subject will excuse the liberty I have taken,

I have the honor to be your Royal Highness's

Most obedient, humble servant,

