# Errata

**Published:** 1799-11

**Authors:** 


					r. 234, 1. 22, Jor " particularly injurious, read " equally deleterious.
P. 284, 1. 3S'ftr " caufe," read " cure."

				

